# Schooling Fish Under Attack Are Not All Equal: Some Lead, Others Follow

**DOI:** 10.1371/journal.pone.0065784

**Published:** 2013-06-12

**Authors:** Stefano Marras, Paolo Domenici

**Affiliations:** IAMC-CNR, Località Sa Mardini, Torregrande, Oristano, Italy; Cajal Institute, Consejo Superior de Investigaciones Científicas, Spain

## Abstract

Animal groups such as fish schools, bird flocks and insect swarms appear to move so synchronously that they have long been considered egalitarian, leaderless units. In schooling fish, video observations of their spatial-temporal organization have, however, shown that anti-predator manoeuvres are not perfectly synchronous and that individuals have spatial preferences within the school. Nonetheless, when facing life-or-death situations, it is not known whether schooling fish react to a threat following a random or a hierarchically-based order. Using high-speed video analysis, here we show that schooling fish (Golden grey mullet, *Liza aurata*) evade a threat in a non-random order, therefore individuals that are first or last to react tend to do so repeatedly over sequential stimulations. Furthermore, startle order is strongly correlated with individual positional preferences. Because school members are known to follow individuals that initiate a manoeuvre, early responders are likely to exert the strongest influence on the escape strategy of the whole school. Our results present new evidence of the intrinsic heterogeneity among school members and provide new rules governing the collective motion of gregarious animals under predator attack.

## Introduction

Group living is a widespread behaviour, observed in many animal taxa [1]. The collective manoeuvres of gregarious animals reflect remarkable coordination, as is commonly observed in fish schools and bird flocks. In fish, collective behaviour has long been assumed to be generated in a self-organized manner in which individuals within the school appear to move in synchrony like an egalitarian *superorganism* [2], [3], [4]. This view has changed in more recent years because high-speed video observations on the spatial-temporal organization of schooling fish have shown that their anti-predator manoeuvres are not perfectly synchronous [5], [6] and behavioural observations have demonstrated that individuals have spatial preferences within the school due to a number of individual attributes [1], [7], [8], [9], [10].

Predator avoidance is considered one of the main advantages of schooling in fish [8], however, very little is known about the behaviour of each school member during anti-predator manoeuvres. Previous studies have mainly investigated the escape response of solitary fish, focusing on locomotor kinematics [11], [12], [13]. Escape responses consist of brief and sudden accelerations in a direction away from the threat and are used by most fish species to avoid predation [14]. One of the main variables affecting prey survival is the timing with which individuals react to an approaching predator [15], [16]. A fundamental component of timing is escape latency, defined as the time interval between the stimulus onset and the first detectable movement of the prey [11]. Short escape latencies, of the order of 10–20 ms, are due to the neural control of a pair of giant neurons present in most fish species (Mauthner cells, [17], [18]), although alternative neural circuits with longer reaction times are known [19]. Previous work has shown that schooling fish tend to have longer latencies than solitary fish, possibly as a strategy for avoiding collisions among neighbouring fish [20]. This suggests that social interactions may be the main factor affecting the timing of the reaction of fish in a school, rather than neuromuscular performance as in solitary fish [21]. While some of the rules underlying the rapid decision-making processes at the base of the coordination of collective manoeuvres in fish and other taxa have been revealed [6], [22], [23], [24], [25], [26], the possibility that leadership-followership rules govern the motion of gregarious animals under predator attack remains to be tested.

Here we investigated the hypotheses that the anti-predator response of a school of fish reflects a non-random startle order, whereby school members that are the first or last to respond to a threat, tend to do so throughout a sequence of stimulations, and that startle order of school members is modulated by positional preferences. These hypotheses were tested in schools of 10 juvenile golden grey mullet (*Liza aurata*). This school size is biologically relevant for many species of nearshore and freshwater fish [9], [27], [28] including mugilids in coastal areas [29], [30].

## Materials and Methods

### Ethic Statement

The fish were held, and the non-lethal experiments were conducted, in accordance with the laws governing animal experimentation in Italy. The IAMC-CNR facility of Oristano, where the fish were held and the experiments performed, is recognised by the Italian Government as a certified facility for fish rearing and ecophysiological experimentation (D.lgs. 116/92, Decreto n° 136/2011-A).

### Animals

A total of 70 juvenile golden grey mullet (*Liza aurata*) [13.4±1.2 cm total length and weighing 14.7±4.5 g (mean±S.D.)] were used. Fish were maintained in a circular tank (150 cm diameter and 50 cm of depth) supplied with recirculated and filtered natural seawater (salinity 29%, temperature 19–20 C°) under natural photoperiod. Fish were fed *ad libitum* twice a week on commercial dry pellets. Feeding was discontinued 2 days before the tests.

### Experimental Set-up and Protocol

**Figure 1:**
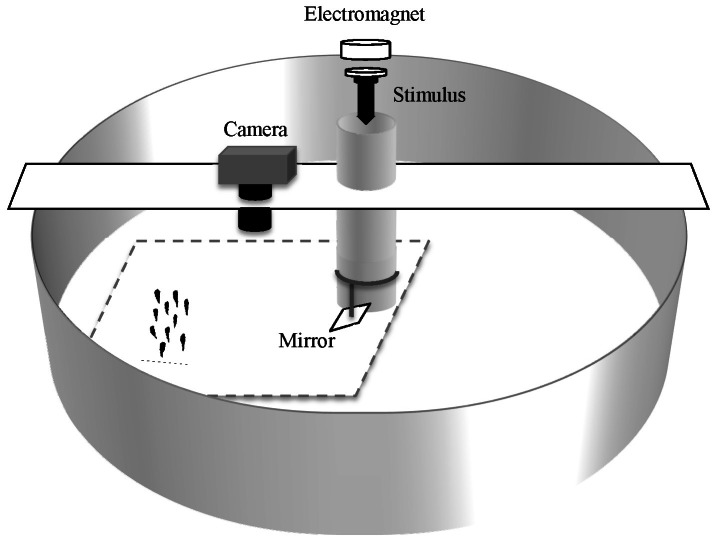
Experimental setup. Experimental tank in which schooling fish were startled by the stimulus released by an electromagnet and recorded using a high-speed camera positioned above the tank. The contact between stimulus and water surface was reflected by a mirror and recorded by the camera. The field of view of the camera is represented by the area delimited by broken lines.

Experiments were carried out in a circular tank (200 cm diameter × 150 cm depth and 36 cm water depth) which was supplied with recirculating seawater at 19–20°C (Fig. 1). Fish were tested in randomly assorted schools of ten individuals. Before the test, fish were marked dorsally, in front of the dorsal fin, using a mix of cyanoacrylate glue and non-toxic white powder insoluble in water (Titanium (IV) oxide, Sigma). Different symbols of approximately 1×0.5 cm were used for each fish so that individuals could be discriminated during the video analysis. Marking took 1 minute for each fish. After marking, fish were placed in a rectangular tank (60×40 cm and 20 cm water depth) and then introduced in the experimental tank all at the same time. They were left undisturbed for 60 minutes of acclimation, after which escape responses were induced by mechanically stimulating the school. The mechanical stimulus was a cylindrical object (10 cm height, 2 cm diameter and weighing 35 g) consisting of a PVC tube with a tapered point, attached to an iron disk in the opposite side. The stimulus was released by an electromagnet from a height of 150 cm above the water surface. To prevent visual stimulation before contact with the water surface, the stimulus was released near the centre of the circular tank, into a vertical opaque PVC tube (10 cm diameter) ending 1 cm above the water surface [31].

**Figure 2:**
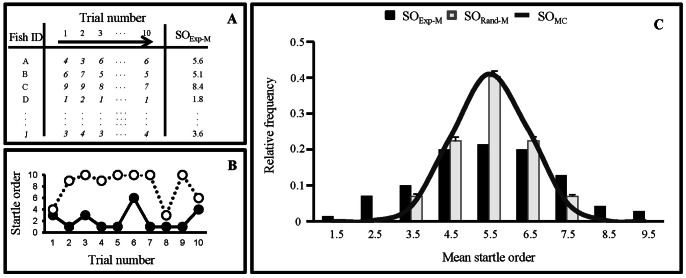
Fish show a non-random startle order. (**A**) Example of an experimental block, defined as a series of ten trials (ten successive stimulations of the same school). Numbers in italics indicate SO of a given fish in a given trial (e.g. in trial #1, fish A is the 4^th^ individual to react). (**B**) Individual fish tended to retain similar startle orders through 10 successive escape responses. Examples of a fast (filled symbols, continuous line) and a slow reacting fish (open symbols, dotted line) within a school. (**C**) The SO_Exp-M_ distribution (black columns, N = 70) showed a statistically wider variance than all of the SO_Rand-M_ distributions [grey columns represent the mean ± S.E. of the ten simulations of SO_Rand-M_ (N = 70 for each simulation)] and the SO_MC_ distribution (dark line, N = 20000).

A high speed camera (Fastec Imaging, Ranger 1000) was positioned directly above the experimental tank and recorded the escape response at 250 Hz (Fig. 1). A moderate circular flow (∼3 cm s^–1^) was induced in the experimental tank by the inflow of the re-circulating filter system. This flow elicited positive rheotaxis, which induced the school to maintain a relative stable position in the tank for the duration of the experiment, allowing stimulation to be delivered consistently to the side of the school (88±20°). To video record the time of the impact between stimulus and the water surface, a mirror inclined at 45° was attached to the end of the vertical PVC tube [32] (Fig. 1). The camera was triggered to record from 1 second before the stimulation to 3 seconds after the stimulation. Each school was stimulated 10 times (hereafter defined as trials, with ten trials constituting a “block”, Fig. 2A), at 10-minute intervals, after which fish were removed from the experimental set-up and measured for length and weight. A total of 7 schools were tested. For some schools, technical problems caused the loss of some trials (a total of 6 trials in 3 schools). Escape sequences were analyzed using Redlake MotionScope PCI (Ver. 2.21.1.).

### Experimental and Random Variables

The mass (M) and fork length (BL) of each fish were used to calculate condition factor (K_f_, 100*M/FL^3^), as an index of the relative stoutness of each individual. Latency (L_e_) was defined as the time interval in ms between the instant when the stimulus broke the water surface and the first detectable escape movement of the fish. If a fish did not react to the stimulus, a latency corresponding to the longest value recorded in our data was assigned to these non-responders. School latency (L_e–s_) was defined as the mean L_e_ of the individuals of a given school for each trial. Distance from stimulus (D_s_) was measured as the distance between the centre of the mechanical stimulus and the centre of mass of the fish measured on stretched-straight specimens [33] (0.4 Length from the tip of the head, [34]). School distance (D_s–s_) was calculated as the mean D_s_ of the individuals of a given school for each trial. The experimental startle order (SO_Exp_) was determined by ranking the individual escape latency (L_e_) within each school for each of the 10 stimulations (trials). SO_Exp_ ranged from 1 (first responder) to 10 (last responder) according to L_e_. Tie events were exceptional (5 events in total). In these cases the fish that were tied were ranked randomly. In the case of a no-response event for a single fish (14 events in total), that fish was ranked last. In cases when multiple fish did not respond (11 events in total), the non-responsive individuals were randomly placed at the end of the ranking. Overall, the proportion of non-responding fish at the individual level when considering all trials was 7%. A mean experimental startle order (SO_Exp-M_) was calculated for each individual through the 10 trials.

**Figure 3:**
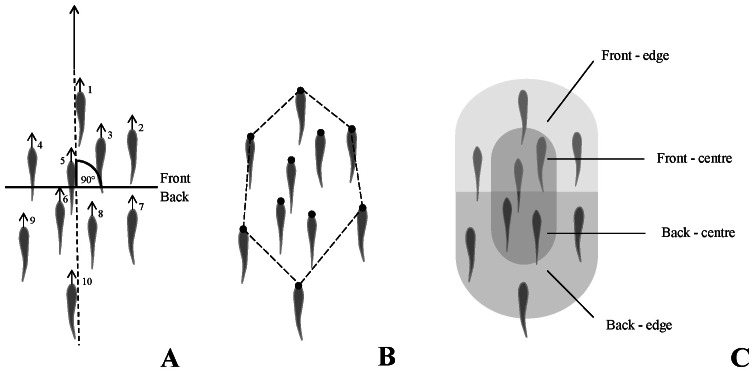
Fish positions within a school. Top view of a school of 10 fish. (**A**) Individuals were numbered according to their position relative to the orientation of the school (O_s_, indicated by the frontal filled arrow). Fish in the first half of the school (position 1 to 5) were considered to be in the front, while fish in the second half of the school (position 6 to 10) were considered to be in the back. (**B**) a fish was considered to be at the edge of the school if its tip of the head was at the vertex of the smallest convex polygon enclosing the entire school (1). (**C**) The four sectors (front-edge, F–E; front-centre, F–C; back-centre, B–C and; back-edge, B–E) were assigned to individuals of the school according to the definitions given for Figures 3A and 3B.

Positional preference was measured, for each individual, as the proportion of times (in % out the ten trials) a fish was in the front (Fp_Exp_) and at the edge of the school (Ep_Exp_), respectively (Fig. 3). In each trial, within-sector SO_Exp_ was determined for each individual by ranking the escape latency within its sector of preference, defined as the sector it occupied most often during the 10 trials. Ties for sector preference were split randomly. Because the number of individuals within each sector varied, within-sector SO_Exp_ data were normalized using ((SO_Exp_−1)/(N−1)) [35], where N represents the number of fish within that sector. Thus, within-sector SO_Exp_ ranged from 0 to 1. For each individual, within-sector SO_Exp-M_ was calculated as the mean of all its within-sector SO_Exp._

A set of random variables was determined. Within each school, for each of the ten trials, ten sequences of random numbers (obtained using Research Randomizer, Royal Psychology Network; www.randomizer.org/form.htm) ranging from 1 to 10 were generated (SO_Rand_). Seven random schools thus generated were equivalent to the seven experimental schools in terms of number of trials as well as missing data. A mean random startle order (SO_Rand-M_) for each individual was calculated as the mean of all its SO_Rand_. A random probability distribution of startle order (SO_MC_) was obtained using a Monte Carlo simulation which generated random permutations. We generated a large number of random data sets using a custom-made Fortran 90 code. We judged SO_MC_ to be stationary when an N of blocks = 2000 was used (i.e. 20000 individuals in 2000 schools composed of 10 fish each), rendering redundant a full analytical computation of all possible permutations (10!^10^). Two sets of distributions of SO simulations based on sorting rules (see Methods S1) were obtained using the same procedure as for SO_Rand-M_ (a simulation of seven 10-trial blocks, repeated 10 times) and SO_MC_ (2000 10-trial blocks). Two sets of distributions of SO simulations based on sorting rules were obtained according to the same scheme as for SO_Rand-M_ (a simulation of seven 10-trial blocks, repeated 10 times) and SO_MC_ (2000 10-trial blocks). Sorting rule A was based on a predetermined circular sequence that followed a fish that was randomly chosen as first responder (see Methods S1). Sorting rule B was based on a first responder chosen at random, followed by responders whose probability of startling was based on the proximity to the previous responders (e.g. fish #9 being proximal to fish #8 and fish #10), (see Methods S1). For each repetition, positions in the front-back (Fp_Rand_) and edge-center (Ep_Rand_) were randomly reassigned using the proportion observed in any given experimental trial. The within-sector SO_Rand_ was derived by randomly reassigning the within-sector SO_Exp_. For each individual, this procedure was applied only within the sector for which they had shown a relative preference during the experimental trials. Ties for sector preference were split randomly. An individual mean random startle order (within-sector SO_Rand-M_) was calculated as the mean of all its within-sector SO_Rand_.

### Statistics

To test whether escape latency was affect by fish body length (BL), condition factor (K_f_) or stimulus distance (D_s_), at the individual level, we ran a multiple regression having the mean L_e_ for each individual as dependent variable, and the individual BL, K_f_, and mean D_s_ as independent variables. To test the effect of K_f_, BL and D_s_ on escape latency at the school level, we ran a multiple regression having the mean L_e–s_ for each school as dependent variable, and D_s–s_, mean BL and mean K_f_, and as independent variables.

Testing whether SO_Exp_ reflects a random order was done in two steps: i) Among-school differences in the variances of SO_Exp-M_ were tested using a Bartlett test. ii) If no differences among schools were found, individuals from different schools were pooled and the variances of SO_Exp-M_ were compared with those of SO_Rand-M_ and SO_MC_ using F-tests. The comparison between SO_Exp-M_ and SO_Rand-M_ was performed ten times, with ten different random simulations.

To determine whether each individual had the tendency to keep a preferential position in the school was done in two steps: i) a Bartlett test was used to test whether the variances of Fp_Exp_ and Ep_Exp_ were independent of school. ii) If no among-school differences were found, the frequency distribution of the pooled Fp_Exp_ and Ep_Exp_ were compared with those of the Fp_Rand_ and Ep_Rand_, respectively, using F-tests.

To test whether SO_Exp-M_ was affected by individual positional preference, an ANCOVA with SO_Exp-M_ as a dependent variable and school (1 to 7), Fp_Exp_ and Ep_Exp_ as independent variables was used. If no effect of school in the ANCOVA was found, data were pooled in order to test the relationship between SO_Exp_ and Fp_Exp_ and Ep_Exp_ using a multiple linear regression.

In order to partition the effect of individual from that of the sector occupied, defined as one of four possible combinations of front-back and edge-centre (i.e. frontal-edge, F–E; frontal-centre, F–C; back-edge, B–E and; back-centre, B–C; Fig. 3C), we tested if the distribution of within-sector SO_Exp_ differed from that of within-sector SO_Rand_. Among-school differences in the variances of within-sector SO_Exp-M_ and within-sector SO_Rand-M_ were tested using a Bartlett test, within each sector. If no differences among schools were found, data from different schools were pooled and the distribution of within-sector SO_Exp-M_ was compared with that of within-sector SO_Rand-M_ for each sector using F-tests.

Parametric statistics were used for all tests, except where conditions of normality (D’agostino test) did not apply. In these cases, non-parametric statistics were applied and the Median-based Levene test was used instead of the Bartlett test. A probability less than 5% (p<0.05) was taken as the limit for statistical significance.

## Results

Fish body length (BL, 13.4±1.2 cm), condition factor (Kf, value 0.6±0.1) or distance from the stimulus [D_s_ at the individual level, (62±18 cm); D_s–s_ at the school level, (62±17 cm)] had no effect on latencies measured at the individual (L_e_) and at the school level (L_e–s_) (Multiple linear regressions; p>0.05 in all cases).

The distribution of SO_Exp-M_ was independent of school (Bartlett test; p = 0.28) and the variance of the pooled SO_Exp-M_ differed significantly from each of ten random simulations (F-test; SO_Exp-M_ *vs.* SO_Rand-M_, p<0.001 in all 10 cases, Fig. 2C), with the experimental data showing a wider range of values (1.8–9.0) than those of their random equivalent SO_Rand-M_ (mean range 3.3–7.6). The pooled SO_Exp-M_ differed significantly from SO_MC_ (F-test; SO_Exp-M_ *vs.* SO_MC_, p<0.001; Fig. 2C). All of the distributions derived from the simulations based on sorting rules are significantly different from the experimental data (F-test; all p<0.05), and not different from SO_Rand-M_ (F-test; all p>0.05). This indicates that the startle order significantly deviates from random in schooling fish, such that individuals that responded early or late in the first trial tended to respond early or late, respectively, in subsequent trials (Fig. 2B).

**Figure 4:**
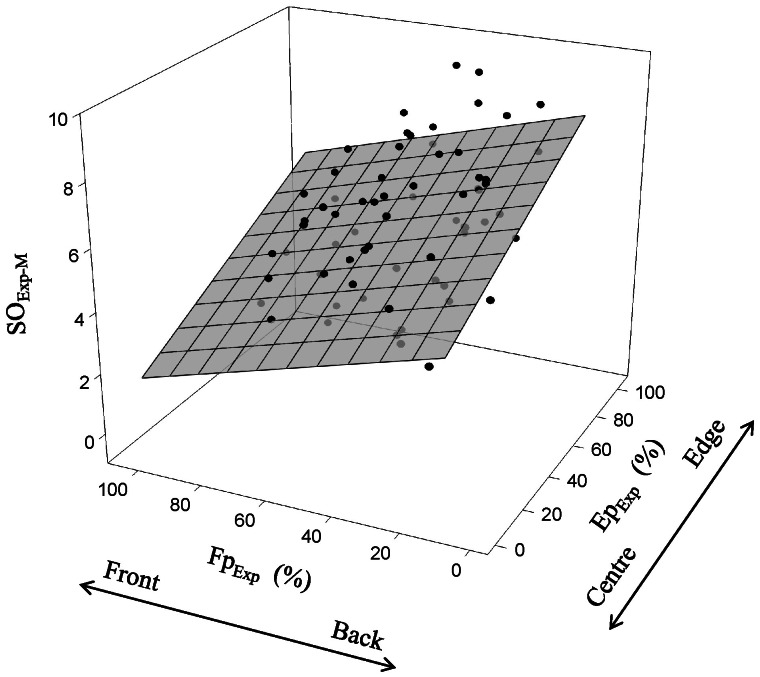
The relationship between mean startle order (SO_Exp-M_) and fish positional tendencies within the school. The mean startle order depended on the positional tendencies of each individual fish at the time of stimulation. The X- and Z-axes correspond to the % of events in which each individual was in the front and at the edge of the school, respectively. Fish in the frontal and central positions react earlier than fish in the back and at the edge of the school. SO_Exp-M_ = 4.78+0.036 Ep_Exp_ − 0.029 Fp_Exp_; p<0.001, R^2^ = 0.38; N = 70.

The distributions of both Fp_Exp_ and Ep_Exp_ were independent of school (Bartlett test; Fp_Exp_, p = 0.92; Ep_Exp_, p = 0.49). The pooled Fp_Exp_ and Ep_Exp_ showed a wider range of values (10–100% and 0–100%, respectively) than those of the Fp_Rand_ and Ep_Rand_ (25–86% and 30–89%, respectively) and their variances differed significantly, indicating individual positional preferences (F-test; Fp_Exp_ *vs.* Fp_Rand_ p<0.001; Ep_Exp_ *vs.* Ep_Rand_ p<0.001). Our results show that both individual SO_Exp_ and SO_Exp-M_ were affected by the position occupied in the school. Individual SO_Exp_ was significantly related to the fish position at the time of the stimulation i.e Front or Back and Centre or Edge (Multiple regression; p<0.001). SO_Exp-M_ was significantly related to both Fp_Exp_ (ANCOVA; p<0.001) and Ep_Exp_ (ANCOVA; p<0.001), independent of school (ANCOVA; p = 0.66), indicating that fish with a tendency to keep frontal and central positions in the school were among the first to escape, while fish that were inclined to keep back and edge positions were among the last (Multiple linear regression, p<0.001; Fig. 4).

The distribution of within-sector SO_Exp-M_ was independent of school (Median-based Levene test; sectors F–E, p = 0.78; F–C p = 0.23; B–E, p = 0.76; B–C, p = 0.40), and their variances of two out of four sectors differed significantly from within-sector SO_Rand-M_ (F-test; F–E, p = 0.16; F–C p<0.01; B–E, p<0.01; B–C, p = 0.54).

## Discussion

The results demonstrate that schooling fish evade a threat in a non-random order, therefore individuals that react early or late tend to do so repeatedly over sequential stimulations. Furthermore, we show that the startle order of each individual fish is modulated by its positional preference.

The finding of non-random startle order is important for elucidating the bases of the collective escape manoeuvres in fish. Predator strikes and prey escapes in fish are typically short lived (in the order of ms) and timing may be a major determinant of survival [14], [15], [16]. In our experiments, while all fish could perceive the stimulus (see Methods S1) the long interval between the first responders (L_e_, 82±30 ms) and those of the rest of the responders (L_e_, 255±190 ms) suggests that most school members could potentially have reacted to the motion of the first responders [36], [37]. Although we cannot establish whether late responders reacted to the stimulus or to their neighbours, the motion of startled members of the school is known to affect the response of other fish. Previous studies have shown that gregarious individuals react both to the predator and to their neighbours [8], [38], [39], [40] and that school members tend to follow neighbours that initiate a manoeuvre [41], [42], [43]. Therefore, because certain individuals react consistently before other school members in successive attacks, they are likely to exert a form of leadership affecting the anti-predator manoeuvres of the whole school.

The range of L_e_ observed in the present study is rather wide (36–1468 ms); most values are beyond those expected for minimal response time [44], [45] and higher than those found in solitary fish of the same species (15–19 ms [34]). Therefore, unlike the variation in escape latency observed in solitary sea bass (*Dicentrarchus labrax*), which was suggested to be caused by differences in neuromuscular performance among individuals [21], the startle order observed here is more likely to be due to behavioural factors. In fish, the riming of escape reactions is influenced by boldness [46], laterality [32] and the perceived risk of predation, e.g. the presence of refuges [47]. In schools, the risk of predation varies in relation to the position occupied [1]; work on schooling chub (*Semotilus atromaculatus*) showed that the risk of predation tends to be higher in the front of the school [48]. This is consistent with the relationship between positional preferences and reactivity found here, i.e. fish at the rear (with a low perceived risk of predation) tend to react later than front fish (with a high perceived risk). However, we found that fish in the centre of the school tend to react sooner than those at the periphery, in contrast with most previous work which found that fish at the edge of the school have a higher risk of predation [1]. Therefore, the perceived risk of predation may not be the only factor affecting the startle order in schooling fish. In addition to the effect of positional preferences, we found that startle order differed from random in two out of four school sectors, suggesting individual tendencies to keep a given startle order within those sectors.

Previous work found that positional preferences can be related to a number of factors such as hunger level [48], behavioural lateralization [49], and metabolic scope [10]. Hunger-related positional preference were minimized here by the standardized feeding protocol. Consequently, the positional preferences observed are likely to be related to individual traits that are repeatable (metabolic scope [50], [51] and inheritable (lateralization [49]). The position occupied by individuals in a group has attracted a lot of attention in the literature, due to the fundamental trade-offs associated with it. Fish in the front were shown to have higher feeding success [1], [7], although they incur higher swimming costs than trailing fish [10], [52]. By demonstrating that positional preferences affect the startle order of fish under attack, the present results provide a novel relationship that can contribute to our understanding of the heterogeneity among the individuals that make up a school.

To conclude, our findings suggest that certain individuals are likely to play a key role on the survival of a whole school under attack. The heterogeneity in the behaviour of schooling fish revealed here can have important ecological implications because of its potential contribution to within and among schools differences in survival during collective anti-predator manoeuvres. The gregarious behaviour of a number of taxa shows similar characteristics to schooling fish in terms of their decision making and anti-predator strategies [1]. It is therefore possible that a non-random pattern of startle order may be a common feature of the coordinated anti-predator manoeuvres displayed by other animal groups.

## Supporting Information

Methods S1 **Additional experiment on the escape reaction in solitary fish and fish in a simulated school and simulation based on sorting rules.**
(DOC)
