# Supplementary material for: Schooling Fish Under Attack Are Not All Equal: Some Lead, Others Follow
Source: PLoS One. 2013 Jun 12;8(6):e65784. doi: 10.1371/journal.pone.0065784 (PMC3680440; doi:10.1371/journal.pone.0065784)
Supplement: Methods S1 — Additional experiment on the escape reaction in solitary fish and fish in a simulated school and simulation based on sorting rules. (DOC) [file pone.0065784.s001.doc]

**Supporting Information:**

**Methods S1**

**Ms: Schooling fish under attack are not all equal: some lead, others follow**

By: Stefano Marras and Paolo Domenici

**Additional experiment on the escape reaction in solitary fish and fish in a simulated school**

In order to test whether or not individual fish, stimulated within the range of stimulus distance used in the experiments, perceived and reacted to the stimulus even in the absence of reactive neighbors, we performed an additional experiment using the same set up as the that used for the primary experiment. We stimulated 8 individual fish, i.e. 4 solitary fish and 4 fish surrounded by neighbors (previously euthanized with cranial concussion, in according with the laws governing animal welfare in Italy, D.lgs. 116/92, and suspended in the water in a typical school shape using transparent threads hanging from the ceiling). The mean D_s_ used in this additional experiment was 107.5±7.26 cm for solitary individuals and 115.7±4.28 cm for individuals in the simulated school), i.e. larger than the maximum D_s_ measured in the experiments (101 cm). In all cases, fish responded to the stimulus, with L_e_ that were within the range of those found in the primary schooling experiments (mean L_e_, 91±13.8 ms for solitary individuals; 87±18.8 ms for individuals in the simulated school). This proves that a fish can perceive and react to the mechanical stimulus, within the range of distances we used in the main experiments, whether it is solitary or in the middle of a non-reactive school.

**Simulations based on sorting rules**

Two sets of distributions of SO simulations based on social rules were obtained base the same scheme as for SO_Rand-M_ (a simulation of seven 10-trial blocks, repeated 10 times) and SO_MC_ (2000 10-trial blocks). The sorting rules used were set as follow: Sorting rule A; Individuals were numbered from one to ten. The first responder was extracted at random out of the ten individuals, then the remaining individuals followed a predetermined series of startle order following the numerical order (1 to 10) in a circular fashion e.g. if the first random extracted individual was number 9, the second responder was the number 10, followed by 1, 2, 3, 4, 5, 6, 7, 8; Sorting rule B; As in sorting rule A, individuals were numbered from one to ten and the first responder was extracted at random. The second responder had a probability to follow the first individual, that decreased according to how close it was, in its number, to the first responder. The percentage of decrease used was 50% at each step. For all subsequent responders, the probability to show a reaction was given by the sum of the probabilities that were due to all previous responders. At each step, the probabilities were rearranged proportionally in order to achieve a sum=100.
